# Regaining Apical Patency with Manual and Reciprocating Instrumentation during Retreatment

**DOI:** 10.22037/iej.v13i3.18020

**Published:** 2018

**Authors:** Rafaela Cristina Trierveiler Paiva, Caroline Solda, Felipe Vendramini, José Roberto Vanni, Flávia Baldissarelli Marcon, Volmir João Fornari, Mateus Silveira Martins Hartmann

**Affiliations:** a * Board Certified in Endodontics by * *Centro de Estudos Odontológicos Meridional* *, Passo Fundo, RS, Brazil* *; *; b *Department of Endodontics at Centro de Estudos Odontológicos Meridional, Passo Fundo, RS, Brazil *

**Keywords:** Apical Patency, Reciprocating, Retreatment, Root Canal Treatment

## Abstract

**Introduction:**

Different techniques have been proposed to help achieving apical patency during endodontic treatment and retreatment. The objective of this *in vitro* study was to compare reestablishment of apical patency in teeth previously subjected to root canal treatment using manual and reciprocating instruments.

**Methods and Materials:**

A total of 40 single-rooted extracted human mandibular incisors were selected and prepared using the Hero 642 sequence to 45/0.02 and obturated using Tagger’s hybrid technique to 1 mm short of the apex. Teeth were divided into two groups according to the type of instrument used to regain patency: group 1, hand K-files and group 2, reciprocating WaveOne Primary files (25/0.08). Fisher’s exact test was used in the statistical analysis.** Result: **In group1, apical patency was regained in 9 of the 20 teeth tested (46%), compared to 20 teeth (100%) in group 2. The difference between the groups was significant (*P*<0.0001).

**Conclusion:**

Our study shows that reciprocating instrumentation is more successful in regaining apical patency in single-rooted, previously treated teeth.

## Introduction

When persistent infection is observed following endodontic therapy, there is the need to perform new cleaning and disinfection of the whole root canal system. Non-surgical endodontic retreatment is associated with several difficulties; in particular, the filling material present in the root canal acts as a mechanical barrier against irrigating solutions, intracanal medication and mechanical cleaning. The complex anatomy of the tooth and root canal system poses further challenges to this process [1].

The patency maneuver consists of penetrating an instrument compatible in size with the real length of the tooth with the aim of rendering the entire root canal free of debris during instrumentation [2]. Regardless of the type of instrument employed (stainless steel manual K-files or nickel-titanium rotary instruments), the patency maneuver may produce different degrees of foramen deformation [3]. The literature presents conflicting results about the importance of achieving apical patency, with some studies pointing out that it is not strictly necessary during endodontic treatment [4, 5]. Similarly, some studies consider cleaning of the apical foramen (*i.e*., after achieving apical patency) as a major prognostic factor of endodontic practice in general and of endodontic retreatment in particular [6]. In addition, maintenance of apical patency has recently been associated with a lower degree of postoperative pain [7].

With the goal of enhancing endodontic retreatment, making it safer, more effective and faster, new techniques and instruments, in particular rotary and reciprocating instrumentation, have been proposed and tested using different assessment methods. With current technological advancements, apical patency can be safely obtained in untreated root canals using reciprocating instruments (WaveOne Primary) [8]. Techniques and instruments have been compared through tooth sectioning, radiography, tomography, and photography, always with a view to fulfilling the primary goal of non-surgical endodontic retreatment, namely, fully removing, or removing as much as possible, the filling material present inside the root canals [9].

**Figure 1:**
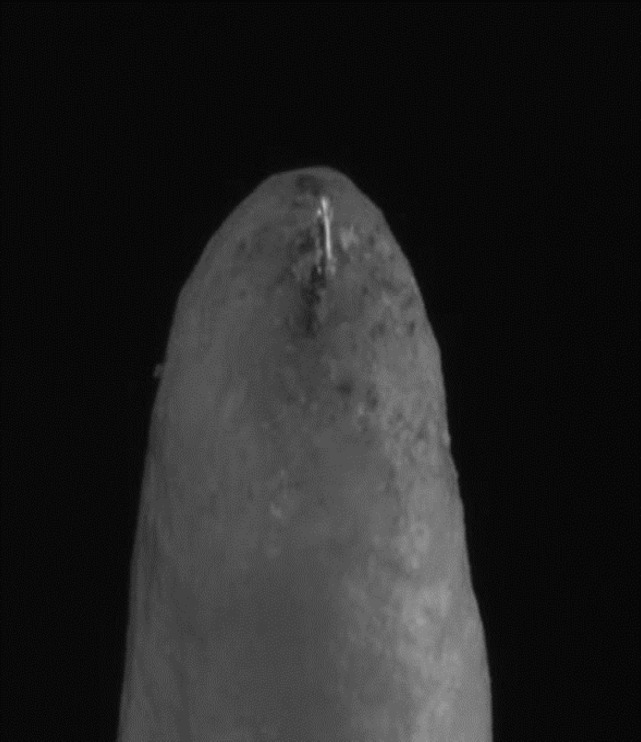
Manual K-File reaching apical patency

In response to the challenges associated with endodontic retreatment, the use of single-file reciprocating instrumentation has become a trend both in the market and in research, with important clinical benefits [10]. However, up to the present moment, no study has been conducted to evaluate the reestablishment of apical patency in endodontically treated teeth. This study was designed to test the use of a reciprocating instrument for that goal. The null hypothesis was that reciprocating instruments would not be more useful than the traditionally used manual K-Files in helping obtain or reestablish apical patency in teeth subjected to non-surgical endodontic retreatment. 

Therefore, the objective of this study was to compare reestablishment of apical patency using manual K-Files and the reciprocating WaveOne system in teeth previously subjected to root canal treatment.

## Materials and Methods

This study was approved as a biorepository research protocol by the Research Ethics Committee of IMED, Porto Alegre, southern Brazil (protocol no. 801.470).

A total of 40 extracted human mandibular incisors with single roots and single canals, measuring between 20 and 22 mm, were used. Sample size was calculated considering a margin of error of 5% and a confidence level of 95%. 

**Figure 2:**
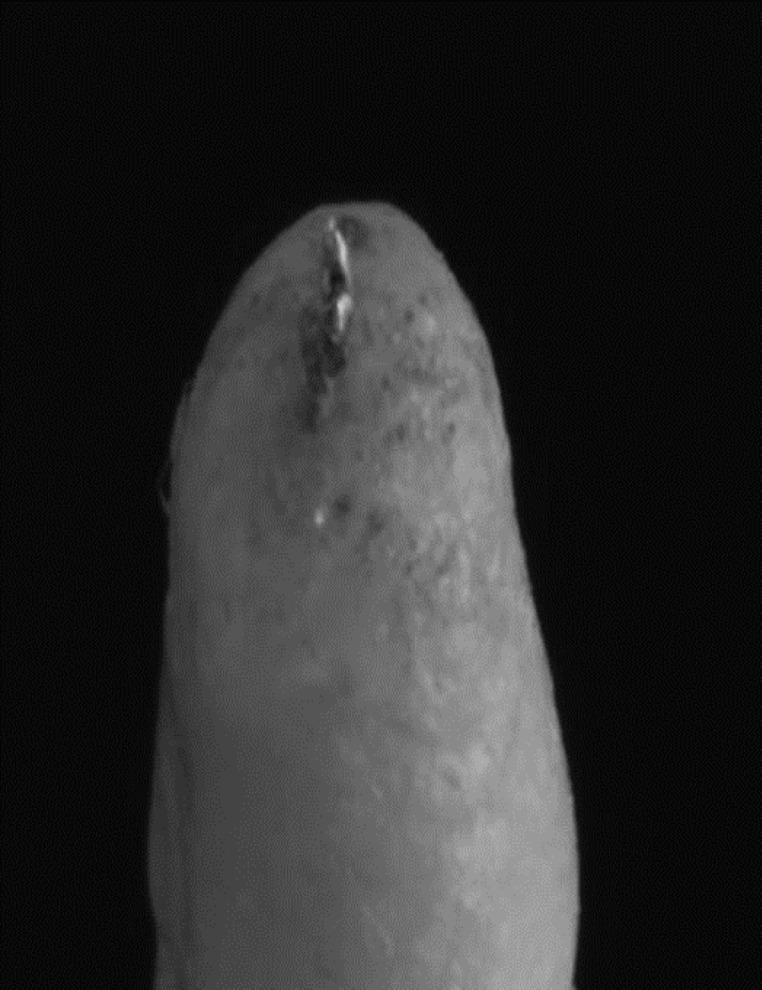
WaveOne Primary file (25/0.08) reaching apical patency

The following exclusion criteria were taken into consideration: teeth with curved canals, calcifications, more than one root canal, previous endodontic treatment and teeth that did not fit the mean length previously determined.

***Preparation***

Teeth were accessed using #1014 KG diamond burs (KG Sorensen^®^, Barueri, SP, Brazil) at high-speed rotation (KAVO, Joinville, Brazil). Once the root canals were located, Gates-Glidden drills #01 to 02 (Dentsply Maillefer, Ballaigues, Switzerland) were used to enlarge the cervical portion of the canal; access was completed using 20/0.06 LA Axxess drills (Dentsply Maillefer, Ballaigues, Switzerland). Root canals were thoroughly irrigated with 2.5% sodium hypochlorite solution (NaOCl), delivered using a disposable plastic syringe (Ultradent Products Inc., South Jordan, USA) and a NaviTip needle (Ultradent Products Inc., South Jordan, USA). Before tooth measurement, root canals were explored using manual #10 K-Files (Dentsply Maillefer, Ballaigues, Switzerland) to determine apical patency.

***Root canal preparation***

Root length was determined by leveling the active tip of a #15 K-File with the apical foramen. Actual working length was established 1 mm short of that measure. The apical foramen was standardized through instrumentation with a #15 K-File to 1 mm short of the working length, followed by Hero 642 instruments (Micromega, Besançon, France), in the following sequence: 020/0.02, 025/0.02, 025/0.04, 030/0.02, 035/0.02, 030/0.06, 040/0.02, and 045/0.02.

All root canals were instrumented to working length using Hero 642 file (Micro-Mega, Besancon, France) size 045/0.02, which was used as the last apical instrument, with irrigation and aspiration at each instrument change. Rotary instruments were coupled to an X-Smart motor (Dentsply Maillefer, Ballaigues, Switzerland), at 350 rpm and 2.8 N/m of torque. When instrumentation was completed, the #15 K-File was once again introduced into the canal until the apical foramen, to confirm canal patency and cleaning. NaOCl 2.5% was used as an adjuvant to root canal treatment. Following preparation, root canals were irrigated with EDTA 17% at pH 7.5 (Extratus Farmácia, Passo Fundo, Brazil), followed by a final flush with NaOCl. Before obturation, root canals were dried using aspiration and absorbent paper points (Dentsply Maillefer, Ballaigues, Switzerland) of diameters compatible with the last apical instrument and with the actual working length. 

Master gutta-percha cones (Dentsply Maillefer, Ballaigues, Switzerland) were then selected for each tooth, once again according to the last apical instrument used and actual working length. All cones were disinfected with NaOCl 2.5% and dried with sterile gauze. Following insertion, fitting of the gutta-percha cone was verified radiographically. 

***Obturation***

Teeth were obturated using Tagger’s hybrid technique. The master gutta-percha cones were placed in the root canals together with Endofill sealer (Dentsply Maillefer, Ballaigues, Switzerland), according to manufacturer’s instructions. Subsequently, accessory cones (Dentsply Maillefer, Ballaigues, Switzerland) were used as necessary until the root canals were completely filled, using the lateral condensation technique in the apical third with a size B finger spreader (Dentsply Maillefer, Ballaigues, Switzerland). Then, the McSpadden NiTi thermocompactor system (Dentsply Maillefer, Ballaigues, Switzerland) was used, at one or two sizes above that of the master cone selected. The compactor was inserted into the root canals at 8000 to 12000 rpm, penetrating to 2 mm short of the actual working length. Following compactor removal, gutta-percha was vertically condensed using a Paiva plugger (SS White, Lakewood, USA) to improve adaptation to the dentinal wall. Excess filling material was removed using cotton balls and alcohol 70^º^GL (Extratus Farmácia, Passo Fundo, RS, Brazil). Teeth were coronally sealed with zinc oxide-eugenol cement (IRM, Dentsply Maillefer, Ballaigues, Switzerland). 

***Filling material removal***

Following obturation of the root canals, teeth were stored in a bacteriological incubator at 37^º^C and 100% humidity for 60 days to allow the filling material to age. 

Before removing the filling material from the cervical and middle thirds of the root canal, one drop of solvent (eucalyptol) was placed at the canal entrance and left to act for 1 min. Filling material was removed using ProTaper retreatment rotary files D1, D2 and D3, always following the same kinematics, at 250 rpm and torque ranging from 1.5 to 2.0 N.cm, coupled to an X-Smart Plus motor, until reaching working length (1 mm short of the apex).

***Apical patency reestablishment***

Apical patency was confirmed visually using two methods: 1) observing the stopper reaching the coronal reference point (cusp tip-1 mm beyond the working length); and 2) observing the tip of the instrument becoming visible in the apical foramen, as shown in Figures 1 and 2.

In Group 1 (*n*=20), apical patency was regained using #15 manual K-Files and the balanced force technique; in group 2 (*n*=20), apical patency reestablishment was performed using single WaveOne Primary files (25/0.08) in reciprocating motion. All attempts to reestablish apical patency were performed by a single calibrated operator. The operator was unaware of the study objective; he was only informed that the study dealt with endodontic retreatment. In one group, the operator was instructed to try to reestablish patency using manual K-files; in the other group, he was instructed to try to reestablish patency using reciprocating files. 

***Data analysis***

All the data collected were recorded and analyzed quantitatively (absolute numbers and percentages) using Fisher’s exact test. Analyses were performed using the Statistical Package for the Social Sciences (SPSS) version 20.0 at a significance level of 0.05.

## Results

In Group 1 (*n*=20), where manual K-files were used, apical patency was regained in nine teeth (46%). In Group 2 (*n*=20), using reciprocating WaveOne Primary files, apical patency was successfully reestablished in all 20 teeth (100%). The difference between the groups was significant (*P*<0.0001) (Table 1).

**Table 1:** Patency results obtained in the two groups

**Groups (N)**	**Apical patency N (%)**	***P*** **-value**
K-Files (20)	9 (46)	<0.0001*
WaveOne Primary (20)	20 (100)	

* *Fisher’s exact test*

## Discussion

Taking into consideration the kinematics of reciprocating instrumentation and previous results on non-surgical endodontic retreatment [11, 12], the aim of the present study was to compare the use of manual K-Files and reciprocating files in regaining apical patency in teeth previously treated endodontically. The results evidence that reciprocating instrumentation is a better alternative to achieve this goal: WaveOne Primary (25/0.08) files successfully allowed to regain patency in all 20 teeth (100%), compared to nine (46%) of the teeth in which manual K-Files were used. 

Our study is the first to assess different instruments in the reestablishment of apical patency, namely, manual instruments used with the balanced force technique [13], and reciprocating instruments used with different anti-clockwise and clockwise angles of rotation (170^º^ and 50^º^, respectively) driven by a motor. The reciprocating instruments were more effective in regaining apical patency, possibly due to the inherent characteristics of this type of movement, which allows the instrument to be more safely inserted in apical direction. Moreover, the design of reciprocating instruments seems to allow better achievement of patency [8]. Finally, the heat treatment to which the M-Wire alloy is subjected results in a safer, more fatigue-resistant instrument [8].

The null hypothesis of the present study was rejected, *i.e.,* reciprocating instruments performed better than manual files in reestablishing apical patency. Also, there were no fractures or any complications in any of the groups, probably because, after each use, instruments were inspected, cleaned and evaluated and immediately replaced whenever any defects were detected. 

The technique used for filling material removal is an important prognostic factor in non-surgical endodontic retreatment, as it represents a new opportunity of biomechanical preparation and root canal disinfection [14]. In this sense, most studies on non-surgical endodontic retreatment show great concern with the complete removal of filling material from the root canal system, often comparing different gutta-percha removal techniques. However, none of the techniques currently available is perfect: all the methods described in the literature leave debris behind [1, 9-12, 14-21]. Carpenter *et al.* [20], in their study on reestablishment of apical patency, compared the use of different solvents to soften gutta-percha and trioxide aggregate (MTA)-based sealer and reported similar results to the ones of the present investigation.

Apical limit and working length determination continue to be controversial topics in endodontics (actual working length at 1 mm short of apex) [2]. However, when retreatment is necessary, there is a critical apical region where a large amount of debris is known to concentrate (necrotic tissues, contaminated filling material, and bacteria). Despite the scarcity of studies investigating apical patency in root canal retreatment [2, 6, 20, 22], there is a consensus that, in this situation, the primary goal is to completely remove (or remove as much as possible) the filling material present in the root canal system, so as to facilitate cleaning [1, 2, 6, 9, 10, 12, 15, 16, 21]. Even though regaining apical patency seems to be equally important in root canal retreatment, this topic has been very rarely addressed. 

Some authors defend the use of a small diameter file, *i.e.*, one that will passively maintain apical permeability, reaching the apical constriction but not enlarging it (K-File). However, appropriately cleaning the apical foramen and regaining apical patency are believed to be essential for a good prognosis following non-surgical endodontic retreatment, precisely because of the potential of this critical apical region to host a higher concentration of debris and bacteria. In most studies conducted to assess filling material removal from root canal walls using tomography, radiography, or tooth sectioning, the apical zone was the one with the poorest cleaning results [1, 9, 11, 12, 14-18, 21]. In this context, if filling material removal to 1 mm short of the apex fails and the canal is contaminated, it seems inappropriate not to conduct full chemical and mechanical cleaning of the apical foramen. Based on the data reported by Negishi *et al. *[6], root canal treatment success rates are lower in teeth where apical patency is not achieved when compared with teeth in which apical permeability is gained. In that study, inaccessible apical constriction increased the risk of treatment failure 5.3 times; whenever both inaccessibility and periradicular lesion were present, the failure rate increased another 4.4 times. 

Few studies have so far assessed whether reciprocating instrumentation is safe and effective in non-surgical endodontic retreatment, probably because this is still a new technology. However, in the few studies found in the literature, reciprocating instrumentation and files (of the two commercially available brands, Reciproc and WaveOne) have been shown to be fast and as effective as manual files or rotary instruments, even though they were not originally designed for filling material removal [11, 12].

## Conclusion

Within the limitations of the present study, the findings showed that reciprocating instrumentation is more successful than manual K-files in regaining apical patency in single-rooted, previously treated teeth.

## Conflict of Interest:

‘None declared’.
